# Sex-Specific Gait Patterns of Older Adults with Knee Osteoarthritis: Results from the Baltimore Longitudinal Study of Aging 

**DOI:** 10.1155/2011/175763

**Published:** 2011-05-09

**Authors:** Seung-uk Ko, Eleanor M. Simonsick, Liz M. Husson, Luigi Ferrucci

**Affiliations:** ^1^Clinical Research Branch, (NIA/NIH), Harbor Hospital, 3001 S. Hanover Street, Baltimore, MD 21225, USA; ^2^Department of Mechanical Engineering, Chonnam National University (CNU), Yeosu 550-749, Republic of Korea

## Abstract

Men and women exhibit different gait patterns during customary walking and may respond differently to joint diseases. The present paper aims to identify gait patterns associated with knee-OA separately in men and women. Participants included 144 men and 124 women aged 60 years and older enrolled in the Baltimore Longitudinal Study of Aging (BLSA) who underwent gait testing at a self-selected speed. Both men and women with knee-OA had lower ankle propulsion mechanical work expenditure (MWE; *P* < .001 for both) and higher hip generative MWE (*P* < .001) compared to non-OA controls. Women with knee-OA had a higher BMI (*P* = .008), slower gait speed (*P* = .049), and higher knee frontal-plane absorbing MWE (*P* = .007) than women without knee-OA. These differences were not observed in men. Understanding sex-specific differences in gait adaptation to knee-OA may inform the development of appropriate strategies for early detection and intervention for knee-OA in men and women.

## 1. Introduction

Knee osteoarthritis (knee-OA) afflicts more than 4 million older US adults [1] and is the most common age-related joint disease that leads to mobility limitations [2, 3]. Gait studies have found that persons with knee-OA have slower gait speed [4–6], smaller knee range of motion [7, 8], and greater medial-lateral knee torque [9–11]. Research has documented important sex-related differences in gait characteristics, including gait speed [12] and mechanical energy usage [13]. Thus, it is conceivable that gait in men and women reacts differently to pathology such as knee-OA. However, full three-dimensional (3D) sex-specific gait studies of adults with knee-OA are lacking. Proper understanding of sex differences in gait patterns in adults with knee-OA is essential for designing appropriate strategies for prevention and intervention to reduce the effect of knee-OA on mobility limitation. The identification of sex-specific gait patterns for OA can be important for early knee-OA detection and for the development of efficient interventions aimed at preventing the clinical progression of knee-OA and its consequences on physical function. 

 We contend that since men and women exhibit different gait kinematics and kinetics during walking at a self-selected speed [12, 13], an analysis of gait patterns that distinguish persons with and without knee-OA done separately for men and women would reveal different patterns. This contention is consistent with studies that have found the etiology of knee-OA to differ between men and women [14]. Understanding sex-specific differences in gait adaptation to knee-OA may inform the development of appropriate strategies for early detection and intervention of knee-OA in men and women. 

## 2. Methods

### 2.1. Participants

 The data reported here are from 268 (124 women) BLSA participants aged 60 to 96 years. After receiving a detailed description of the study and consenting to participate, participants were assessed in the BLSA Gait Laboratory between January 2008 and April 2009. The BLSA protocol was approved by the MedStar Health Research Institute's Institutional Review Board (Baltimore, MD). Combined information from the questionnaire, physical exam, and X-ray was used to adjudicate knee-OA diagnosis according to an algorithm modeled on the American College of Rheumatology (ACR) diagnostic classification criteria for knee-OA [15]. Morning stiffness was ascertained by highly trained nurse practitioners who used the following standard questions: “on most days, in the past 12 months, did you have morning stiffness in either of your knees?” The nurse practitioners also performed a standardized physical exam to identify knee abnormalities such as crepitus, tenderness, and effusion. A posterior-anterior knee X-ray was performed in a standardized fixed flexion position (Siremobile Compact, Siemens, New York) to establish the presence of osteophytes. Briefly, participants with at least 2 of the 4 following clinical findings: crepitus, tenderness, osteophytes, and effusion are classified as having knee-OA. Participants who did not have hip or knee prosthesis, severe joint pain, history of stroke, or Parkinson's disease, and who could follow instructions and safely complete customary walking tasks unaided in the gait lab were included in this study. Participants with a body mass index (BMI) over 40 were excluded because of technical difficulties positioning pelvic markers during the gait analysis.

### 2.2. Gait Measurement

Procedures for the gait analysis performed in our laboratory have been described previously [16, 17]. Briefly, participants were outfitted with 20 reflective markers placed on anatomical landmarks: anterior and posterior superior iliac spines, medial and lateral knees, medial and lateral ankles, toe (second metatarsal head), heel, and lateral wands over the midfemur and midtibia. To avoid errors in hip joint calculations due to excessive adipose tissue of overweight and obese participants, a waist wrap was used in the pelvic area, and the distance between the left and right anterior superior iliac spines (ASIS) was measured manually. A Vicon 3D motion capture system with 10 digital cameras (MX-T40, MX Giganet, Oxford Metrics Ltd., Oxford, U.K.) measured the 3D locations of all landmark markers of the lower extremity segments (60 Hz sampling frequency). During gait testing, ground reaction forces were measured with three staggered AMTI force platforms (Advanced Mechanical Technologies, Inc., Watertown, MA, USA; 1080 Hz sampling frequency). 

After all markers were positioned on the skin or nonreflective tight-fitting spandex tights, participants were asked to walk along a 10-meter walkway at a self-selected speed. Participants were not informed about the presence or location of the force platforms on the walking path. Trials were performed until at least 3 complete gait cycles from the left and right sides with full foot landing on the force platform were obtained. The raw coordinate data of marker positions were digitally filtered with fourth-order zero-lag Butterworth filter with a cutoff at 6 Hz.

### 2.3. Data Processing

Kinematic and kinetic gait parameters measured and calculated using our gait laboratory protocol have been described in detail elsewhere [16]. Briefly, mechanical joint powers of lower extremity rotations in the sagittal plane and frontal plane were calculated by using Visual3D (C-motion, Inc., Germantown, MD, USA). The Bell pelvic model (using the left and right ASISs and PSISs) was used for hip joint calculations [18]. Inertial properties of lower segments were estimated from anthropometric measurements (height and weight) and landmark locations [19]. Based on kinematic measurements, ground reaction forces, and the paradigm of inverse dynamics, gait parameters in kinetics, including joint moment and power were calculated. Mechanical work expenditures (MWEs) were calculated by numeric integration of mechanical joint powers during the stance period using custom made software written in MATLAB (MathWorks, Inc., Natick, MA, USA). To dissect functional differences of MWE in generative and absorptive modes, joint mechanical powers in positive (generative) and negative (absorptive) modes were integrated separately. Spatiotemporal parameters including gait speed, stride length, and stride width were calculated in bundle by Visual3D, and they were manually checked by a technician using custom-made software written in MATLAB.

### 2.4. Statistical Analysis

Statistical analysis was performed using SAS 9.1 Statistical Package (SAS Institute, Inc., Cary, NC, USA). Data are reported as means and standard errors. Cross-sectional comparisons of gait parameters between participants with and without knee-OA were performed using general linear models (GLM) for men and women separately. Associations between age and each gait parameter were examined separately for men and women: the beta (*β*) values for those models represent the averge change estimated in the dependent variable associated with a one unit change in the independent variable. The associated *P*-values test the null hypothesis that the beta value is equal to zero. An interaction term (knee-OA∗age) was included in all models to test the hypothesis that the effect of age on gait was different in participants with and without OA. All analyses were adjusted for gait speed (except gait speed itself), weight, and height. Statistical significance was defined as a *P* value less than  .05.

## 3. Results

Of the 144 men and 124 women included in the study, 29 (20%) men and 31 (25%) women had knee-OA. Participant characteristics are summarized in Table 1. Women with knee-OA had a body mass index (BMI) higher than those free of knee OA, but such a difference was not found in men. Comparisons of spatiotemporal gait parameters are summarized in Table 2. In women with knee-OA but not in those free of knee-OA, older age was associated with slower gait speed and wider stride width. 

Kinematic and kinetic gait parameters in the sagittal and frontal planes are summarized in Tables 3 and 4, separately for men and women. Men and women with knee-OA commonly had a lower ankle peak joint moment in the sagittal plane compared with those without knee-OA. Women with knee-OA walked with a higher hip peak joint moment in the sagittal plane and with higher hip and knee peak joint moments in the frontal plane compared with women without knee-OA. In both men and women, ankle generative MWE in the sagittal plane was lower and knee absorptive MWE in the sagittal plane was higher for those with knee-OA. In women but not in men, knee absorptive MWE in the frontal plane was higher in those with knee-OA. Hip generative MWE was higher in the sagittal and frontal planes in men with knee-OA and in the frontal plane alone for women with knee-OA compared with counterparts free of knee-OA. Ankle absorptive MWE in the sagittal plane was higher for men with knee-OA than those without knee-OA. Other findings restricted to women only include higher ranges of motion for the hip in the sagittal plane and frontal plane in those with knee-OA.

**Figure 1:**
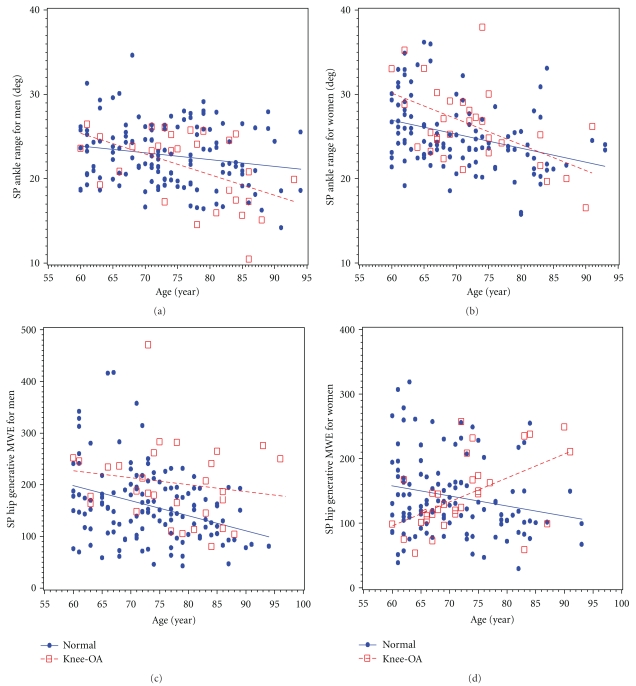
Ankle range of motion in the sagittal plane (SP) for men (a) and women (b) with and without knee-OA by year of age. Hip generative mechanical work expenditure (MWE; J/kg∗1000) in the sagittal plane (SP) for men (c) and women (d) with and without knee-OA by year of age.

Examining the interaction term for knee-OA status and age (knee-OA∗age) revealed that men with knee-OA had steeper age-associated decline in ankle range of motion compared to men without knee-OA (Figure 1(a)) while women with knee-OA had a steeper age-associated increase in hip generative MWE (Figure 1(b)) in the sagittal plane compared to women without knee-OA.

## 4. Discussion

Consistent and unique gait patterns characterizing knee-OA during walking at a self-selected speed were identified for men and women separately. Partially supporting our preliminary hypothesis, we observed that gait patterns associated with knee-OA for men and women while sharing several common characteristics also showed significant differences. Higher BMI with knee-OA [20] was only evident for women. Most differences emerged in relation to the kinematics and kinetics of the lower extremity during normal pace walking. 

Roles of hip musculature in gait have been reported with respect to trunk stabilization [21] and knee-OA [10, 22, 23]. In this study, men and women with knee-OA exhibited slightly different deviations in hip kinematics and kinetics from their counterparts free of knee-OA. It is important to note that findings specific to hip generative MWE in the sagittal plane, which was higher for men and older women with knee-OA suggest that knee-OA-related gait patterns of hip energetics are age-associated for women, but independent of age in men. Thus, knee-OA-specific hip activities may be similar for men and women in old age, but quite different at younger ages (Figure 1(b)). These findings support the notion that gait adaptation to knee OA is different in men, and women and that it is also conditioned by the specific effects of age. Whether these results are due to different age of onset of clinical knee OA is an interesting question that should be addressed in future studies, possibly from a longitudinal perspective. 

Notably, lower knee range of motion which has been consistently reported in previous studies as a characteristic of a knee-OA-related gait pattern [7, 8, 24] was not evident in this study. This may be due to the relatively low symptom burden of BLSA participants at the time of their clinic visit and the general exclusion of persons with severe joint pain during walking from gait lab testing. Nevertheless, the higher absorptive knee MWE in the sagittal plane, consistently observed for both men and women with knee-OA in this study, may directly explain knee joint symptoms. Meanwhile, higher frontal-plane knee kinetics (assessed as peak joint moment and absorptive MWE in this study), considered a risk factor for knee-OA [9, 10, 25], were consistently seen in women only.

Both men and women with knee-OA exhibited lower ankle kinetic activity compared to their counterparts without knee-OA; yet, ankle range of motion which tends to vary systematically with ankle kinetics did not vary with knee-OA status in women and relatively younger men. Thus, younger men and women of all ages with knee-OA appear to have lower efficiency in ankle energy generation, which may explain the observed sex- and age-specific gait characteristics in persons with knee-OA.

Except for hip generative MWE, gait parameters in the frontal plane revealed knee-OA-specific gait patterns only in the women where those with knee-OA showed higher frontal-plane hip and knee activity compared to participants without knee-OA as seen in higher range of motion, higher peak joint moment, and higher generative MWE. In knee-OA women, higher frontal-plane joint moment within constrained knee rotation might cause higher absorbing mechanical energy (36%) of the knee joint in the frontal plane, previously reported as a risk factor for knee-OA [9, 10, 25]. 

 Due to the cross-sectional nature of this study, identifying causality in the association between gait patterns and the prevalence of knee-OA was not possible; thus, discriminating whether the observed associations are causes or effects of knee-OA was not feasible in this study. The BLSA is currently collecting longitudinal gait data which will allow observation of transitions from the normal state to knee-OA. 

In conclusion, findings of the present study extend the existing evidence of knee-OA related gait patterns to sex-specific knee-OA gait patterns. Previously reported knee-OA characteristics of slower gait speed and higher frontal-plane knee kinetics were found in this study, but only for women. In addition, knee-OA-related gait patterns had different age associations for the hip and ankle joints in men specifically. Unique gait characteristics of men and women with knee-OA raised in this study suggest various specific gait patterns as possible risk factors for knee-OA that differ by sex and age. If these sex-specific knee-OA gait patterns can be confirmed in longitudinal studies, clinical diagnostic criteria for early detection of knee-OA may become more precise and allow for more efficient and targeted intervention.

## Figures and Tables

**Table 1:** Participants characteristics.

Variables	Sex	No-OA *N* = 268 (women, *N* = 124)	Knee-OA *N* = 60 (women, *N* = 31)	Comparison, *P* value
Age, years	Men	74	77	.101
	Women	71	72	.255
Height, m	Men	1.73	1.74	.495
	Women	1.62	1.60	.119
Weight, kg	Men	81.78	81.04	.782
	Women	69.56	74.62	.068
BMI, kg/m^2^	Men	27.15	26.66	.497
	Women	26.36	28.97	.008

BMI: body mass index.

**Table 2:** Spatiotemporal gait parameters in men and women with and without knee-OA.

Spatiotemporal gait parameters	Sex	No-OA *N* = 268 (women, *N* = 124)			Knee-OA *N* = 60 (women, *N* = 31)			Mean comparison	Age-association comparison (OA∗age)
		Mean	*β*	*P* value	Mean	*β*	*P* value	*P* value	*P* value
Speed*, m/s	Men	1.13	−0.010	<.001	1.11	−0.010	<.001	.477	.980
	women	1.12	−0.012	<.001	1.06	−0.014	<.001	**.049**	.445
Stride length**, m	Men	1.24	−0.003	.005	1.23	−0.005	.007	.722	.322
	women	1.16	−0.003	<.001	1.19	−0.005	.007	.102	.377
Cadence**, steps/min.	Men	109.51	0.252	.012	110.11	0.385	.018	.729	.442
	women	114.46	0.344	<.001	112.06	0.473	.020	.094	.531
Stride width**, cm	Men	11.31	0.026	.330	11.55	0.090	.004	.626	.081
	women	10.07	0.016	.576	10.36	0.109	.010	.550	**.041**

*β*: estimated coefficient for age association.

*Adjusted by age, height, and weight.

**Adjusted by gait speed, age, height, and weight.

**Table 3:** Gait kinematic and kinetic parameters in the sagittal plane for men and women with and without knee-OA.

Gait parameters in the sagittal plane	Sex	No-OA *N* = 268 (women, *N* = 124)			Knee-OA *N* = 60 (women, *N* = 31)			Mean comparison	Age-association comparison (OA∗age)
		Mean	*β*	*P* value	Mean	*β*	*P* value	*P* value	*P* value
Range of motion, degree*									

Hip	Men	39.56	−0.124	.005	39.57	−0.048	.453	.989	.284
	Women	38.82	−0.074	.116	40.68	−0.163	.141	**.017**	.437
Knee	Men	54.47	−0.153	.002	53.05	−0.181	.144	.129	.827
	Women	54.55	−0.189	<.001	54.37	−0.220	.163	.838	.847
Ankle	Men	22.79	−0.049	.220	21.48	−0.217	<.001	.075	**.013**
	Women	25.20	−0.149	.001	26.73	−0.309	<.001	.065	.098

Peak joint moment, N·m/kg									

Hip	Men	1.14	−0.003	.207	1.23	−0.001	.800	.061	.621
	Women	1.15	0.001	.715	1.29	−0.008	.165	**.011**	.134
Knee	Men	0.67	0.006	**.002**	0.67	−0.002	.437	.950	**.012**
	Women	0.63	0.002	.370	0.68	−0.002	.536	.165	.297
Ankle	Men	1.28	0.01	<**.001**	1.12	0.005	.238	**.002**	.419
	Women	1.20	0.001	.712	1.00	0.014	.007	<**.001**	**.023 **

Total generative MWE**, 1000* J/kg									

Hip	Men	156.55	−0.980	.093	210.14	0.761	.542	<**.001**	.193
	Women	137.31	−0.402	.513	155.38	4.951	<.001	.117	<**.001**
Knee	Men	101.40	−0.786	.086	104.27	−0.776	.263	.774	.990
	Women	84.51	0.560	.290	92.01	−1.089	.190	.412	.072
Ankle	Men	208.63	1.478	.008	158.78	−0.388	.684	<**.001**	.074
	Women	220.01	−0.538	.443	174.69	1.542	.089	<**.001**	.054

Total absorptive MWE**, 1000∗J/kg									

Hip	Men	240.65	−1.49	.096	215.88	−1.58	.362	.151	.960
	Women	267.25	0.778	.466	311.07	−8.14	.001	.061	<**.001**
Knee	Men	167.86	−0.763	.347	197.60	−2.601	.020	**.043**	.148
	Women	167.15	−0.071	.942	225.16	−1.679	.308	**.002**	.370
Ankle	Men	136.22	0.023	.956	153.81	0.140	.868	**.034**	.899
	Women	126.90	−0.535	0.248	130.86	2.470	.044	.660	**.018**

MWE: mechanical work expenditure; *β*: estimated coefficient for age association.

*Adjusted by gait speed, age, height, and weight.

**Adjusted by gait speed, age, and height.

**Table 4:** Gait kinematic and kinetic parameters in the frontal plane for men and women with and without knee-OA.

Gait parameters in the fontal plane	Sex	No-OA *N* = 268 (women, *N* = 124)			Knee-OA *N* = 60 (women, *N* = 31)			Mean comparison	Age-association comparison (OA∗age)
		Mean	*β*	*P* value	Mean	*β*	*P* value	*P* value	*P* value
Range of motion, degree*									

Hip	Men	9.26	−0.092	<.001	9.14	−0.039	.257	.785	.158
	Women	9.80	−0.096	<.001	11.17	−0.057	.402	**.005**	.558
Knee	Men	10.32	−0.093	.037	11.02	−0.209	.007	.394	.154
	Women	9.27	−0.025	.540	9.35	−0.018	.863	.916	.950
Ankle	Men	8.98	−0.036	.172	8.23	−0.144	.016	.170	.076
	Women	10.16	−0.071	.027	9.29	−0.227	.008	.181	.073

Peak joint moment, N·m/ kg									

Hip	Men	0.83	−0.005	<**.001**	0.86	−0.003	.112	.173	.221
	Women	0.84	−0.005	<**.001**	0.91	0.001	.896	**.005**	**.036**
Knee	Men	0.46	−0.001	.596	0.48	0.003	.216	.355	.159
	Women	0.38	−0.002	.097	0.47	−0.002	.448	<**.001**	.938
Ankle	Men	0.17	0.001	.822	0.17	0.001	.757	.456	.757
	Women	0.16	−0.001	.142	0.17	0.001	.559	.329	.254

Total generative MWE**, 1000*J/kg									

Hip	Men	56.22	−1.666	<.001	75.24	−1.743	<.001	**.002**	.887
	Women	67.29	−1.851	<.001	85.43	−0.874	.207	**.002**	.158
Knee	Men	12.54	−0.016	.857	12.64	0.078	.624	.958	.572
	Women	9.90	−0.172	.006	11.81	−0.216	.071	.141	.706
Ankle	Men	9.25	0.084	.269	10.21	0.327	.163	.503	.324
	Women	10.71	0.062	.551	11.02	0.758	.055	.925	.079

Total absorptive MWE**, 1000* J/kg									

Hip	Men	53.72	−0.302	.110	54.22	−0.242	.569	.902	.894
	Women	41.96	0.082	.734	48.84	0.843	.150	.143	.212
Knee	Men	18.42	0.023	.851	21.67	−0.241	.110	.191	.147
	Women	15.99	0.057	.497	21.34	−0.098	.660	**.007**	.473
Ankle	Men	15.52	−0.210	.012	16.83	0.264	.466	.525	.201
	Women	17.16	−0.315	.003	20.13	0.476	.320	.281	.125

MWE = mechanical work expenditure; *β*: estimated coefficient for age association.

*Adjusted by gait speed, age, height, and weight.

**Adjusted by gait speed, age, and height.
